# “It’s sad, and I want to go back to how things were before”: a qualitative study of young people’s experiences of living with long COVID

**DOI:** 10.1136/bmjpo-2025-004167

**Published:** 2026-05-11

**Authors:** Kajsa Järvholm, Maria Bygdell, Johan Dreifaldt, Elin Åkesson, Tove Lundberg

**Affiliations:** 1 Department of Psychology, Lund University, Lund, Sweden; 2 Department of Paediatrics, Skåne University Hospital, Malmö, Sweden; 3 Department of Internal Medicine and Clinical Nutrition, Institute of Medicine, Sahlgrenska Academy, University of Gothenburg, Gothenburg, Sweden

**Keywords:** COVID-19, Qualitative research, Adolescent Health

## Abstract

**Background:**

Long COVID is a newly recognised condition that also affects children and young people (CYP). However, little is known about how it impacts their daily lives and well-being from their own perspective. This study aimed to explore the lived experiences of CYP living with long COVID.

**Methods:**

Swedish-speaking CYP who developed long COVID before the age of 18 years were invited to participate in semistructured interviews. Between February and September 2024, seven CYP (four girls and three boys) participated (mean interview duration=51 min). Data were analysed using reflexive thematic analysis to identify codes and then themes with related subthemes.

**Results:**

Two main themes were generated. The first main theme ‘I am stumbling in the dark: Living with unpredictability and limitations’ covers how CYP were affected by the consequences of long COVID. The subtheme ‘How do I live and function now?*’* illustrates how the CYP tried to navigate the disabling and yet fluctuating symptoms, and the subtheme ‘Who am I now?’ represents the identity-related consequences. The second main theme ‘Navigating responses from others to find support’ illustrates how others have responded to their symptoms and how these responses were perceived by the CYP. The subthemes ‘Stop being sceptical!’, ‘Just try to help me!’ and ‘Accept and validate me!’ illustrate interactions perceived as invalidating or disrespectful, what has been experienced as helpful or unhelpful and the kinds of support adolescents have valued.

**Conclusions:**

Long COVID negatively impacted the CYP’s lives, affecting their relationships, education, leisure activities and sense of identity. Dismissive and sceptical attitudes from professionals and peers substantially increased the burden, whereas encountering acceptance and knowledgeable professionals facilitated coping with long COVID.

**WHAT IS ALREADY KNOWN ON THIS TOPIC**

- Long COVID affects children and young people (CYP), but little is known about their own lived experiences and how the condition impacts their daily lives.

WHAT THIS STUDY ADDS

- This study demonstrates that long COVID is experienced by CYP as a disabling and unpredictable condition that impacts relationships, education and identity. CYP emphasise the importance of healthcare professionals listening, validating their experiences, and offering meaningful support.

**HOW THIS STUDY MIGHT AFFECT RESEARCH, PRACTICE OR POLICY**

- Affected CYP call for compassionate care and support from professionals with knowledge of the condition, to help them navigate the uncertainty of living with long COVID.

## Introduction

 In March 2020, the WHO declared COVID-19 a pandemic.^1^ Already during the first few months, reports emerged of patients with persistent symptoms with substantial impact on daily functioning, after the acute disease.^2^ Initial findings primarily concerned adults, but it soon became evident that children and young people (CYP) could also be affected.^3 4^ Although symptom profiles overlap, fatigue and breathlessness being common in both adults and CYP, adults more frequently report brain fog and musculoskeletal pain, whereas headaches and sleep disturbances are more prevalent among CYP.^5^ There is still no consensus regarding prevalence of the condition,^6^ and a systematic review of long COVID in CYP found a very high variability in the proportion affected (2–70%).^7^

In addition to evaluating the prevalence, risk factors, aetiology, prevention and treatment of long COVID, it is essential to understand the lived experience and perceived health of affected patients to provide better care. There are some qualitative studies on how long COVID is experienced by adults. In a systematic review and meta-synthesis, compiling findings from 12 studies, it was shown that adults living with long COVID experience complex physical health problems but also substantial psychosocial challenges, such as a changed identity and social isolation.^8^

A chronic illness can have an even more disruptive impact in CYP’s lives, as both social and educational development can be interrupted.^9 10^ The identity formation can also be challenged.^11^ According to Article 12 of the Convention on the Rights of the Child, children have the right to be heard,^12^ but research on CYP’s lived experience with long COVID remains limited. The few published qualitative studies on long COVID during childhood and adolescence have shown that long COVID is experienced as severely impairing and impacting almost all aspects of CYP’s lives.^13^,^18^ Parents witness that it is difficult to get the right support from healthcare,^19^ and CYP with long COVID prefer to have the care delivered by clinics specialised in their condition with staff being emphatic and knowledgeable, yet flexible.^13^

Most qualitative studies on CYP’s experiences of long COVID to date have combined data from both CYP and their parents, meaning that the unique perspective of CYP themselves has often been less distinctly explored. Our study seeks to complement and extend previous research by focusing exclusively on CYP’s own voices and lived experiences,^20^ thereby contributing to a nuanced understanding of long COVID from their perspective. The aim was to explore CYP’s self-described experiences of long COVID from a holistic perspective, providing further opportunity for their voices to be heard.

## Methods

### Design

To explore and give voice to the lived experiences of CYP with long COVID, we conducted an interpretive qualitative interview study. Data were generated through semistructured, open-ended interviews and analysed using reflexive thematic analysis.^21^ The study was grounded in a critical realist perspective, which views participants’ accounts as meaningful descriptions of experience shaped by material, social and contextual conditions.

Data collection was planned with common long COVID symptoms, such as fatigue, in mind. Participants could therefore choose between digital and in-person interviews, and they were not asked to provide feedback on transcripts or analyses, a procedure sometimes used in qualitative research.

### Patient and public involvement statement

This study grew out of the specific experiences of clinical work and research involving different interactions with patients and organisations. KJ (a female clinical paediatric psychologist and associate professor) has been involved in setting up a multidisciplinary team for CYP with post-infectious disease at the paediatric clinic at Skane University Hospital. TL (a non-binary clinical psychologist and associate professor specialised in qualitative methodologies in psychology) has been an active patient representative for the Swedish Covid Association and had maintained an ongoing dialogue for 2 years with patients, parents, healthcare professionals and researchers. MB (a female associate professor in epidemiology) has had repeated dialogues with patient representatives during her work with epidemiological studies on long COVID. The patient association actively helped with recruitment of participants for this study and will assist in disseminating the findings.

### Recruitment

Recruitment took place between February and September 2024, and information about the study was posted on social media and the Swedish Covid Association’s website and in two paediatric clinics (purposive sampling). Interested participants could register via a digital form and were then contacted to schedule an interview. They were informed that the research aimed to improve services to CYP living with post-infectious diseases.

### Participants

The primary inclusion criterion was long COVID onset before the age of 18 years. Long COVID was defined according to the WHO criteria, namely persistent symptoms lasting at least 2 months and occurring at least 3 months after acute COVID-19.^22^ CYP aged 12–21 years were eligible. The lower age limit was set to increase the likelihood that participants could engage meaningfully and independently in the interviews, while the upper limit allowed inclusion of those who developed long COVID in late adolescence. Participants needed to be able to conduct the interview in Swedish.

10 CYP showed interest by registering at the digital form. However, one adolescent did not respond to the researchers’ attempts to establish contact, one experienced a worsening of their long COVID and did not have the energy to take part and one had developed long COVID after the age of 18.

### Data collection

The interview guide was developed in line with literature on semistructured interviews and was informed by prior research.^23^ Specific questions concerned experiences and consequences of symptoms, as well as experiences of support. To give the CYP ample space to share what was important to them, the questions in the interview guide were formulated in an open-ended manner where the interviewer would prioritise attending to what the young person said. The interview guide was reviewed after the first completed interview and deemed adequate, and no changes were made.

All interviews were conducted online via Zoom by either JD (male) or EÅ (female). They were at that time in the final stage of training to become clinical psychologists and now hold Master of Science degrees. Both share an interest in paediatric psychology and qualitative methodology. The interviews lasted between 29 and 64 min (mean=51 min). In two interviews, a parent was present for support after the participant expressed this preference at the beginning of the interview. The accompanying parent was informed that the study focused on the CYP’s perspective and that no questions would be directed to them.

### Qualitative analysis

Interviews were audio-recorded, transcribed verbatim and de-identified. In the presentation of the results, all the participants were given pseudonyms. In line with reflexive thematic analysis, we judged the dataset to be adequate for our analytic aims through iterative theme development across the full dataset and the information richness of the interviews.^21^

The inductive critical realist reflexive thematic analysis was conducted in six phases.^21^ After familiarising themselves with the data, JD and EÅ coded the interviews sequentially in NVivo (phases 1–2). Codes in this phase included, for example, Symptoms, Impact of symptoms on school, School adjustment, Self-perception, Distrust from healthcare providers, Desire to be believed and Family support. The codes were then clustered into candidate themes (phase 3). This was an iterative process involving KJ and TL as well, where their clinical and qualitative expertise and knowledge of long COVID helped develop the analysis reflexively. JD and EÅ drafted the initial version of the themes (phase 4) and included the main themes ‘No longer being enough’, ‘Groping in the dark’ and ‘See me, hear me, believe me”. The thematic structure was further developed by KJ and TL to highlight the aim of giving voice to the participants and offer a holistic perspective of their lived experiences.^20^ The final draft was then revised and approved by the entire research team (phases 5–6).^21^ In writing up the results, the COREQ checklist was consistently used to ensure transparent and comprehensive reporting.^24^

Throughout the analysis, we engaged in reflexive discussions and considered alternative interpretive possibilities. In keeping with our aim of ‘giving voice’, we prioritised participants’ meanings and language in the development and presentation of themes. Still, we acknowledge that theme development is an interpretive process also shaped by our clinical and advocacy engagements. For example, all involved researchers share the position that long COVID is a real and significant condition and that CYP deserve high-quality care where their symptoms are taken seriously.

## Results

The participants (four girls and three boys) were between 14 and 20 years old (mean age=16 years) at the time of the interview and had learnt about the study via the Swedish Covid Association’s website (n=4) or social media (n=3). Four of the CYP were in lower secondary school, one was in upper secondary school and two had graduated. The average age at the onset of long COVID was 14 years (range 11–17 years). The CYP did not know exactly when the acute illness transitioned into long COVID. The participants had lived with long COVID for a minimum of 6 months and a maximum of 4 years. All still had symptoms. Most reported symptoms were fatigue (reported by all) and headaches. Other current symptoms included nausea, dizziness, cognitive impairment, pain, fever, tachycardia and shortness of breath, each occurring in at least two participants. Two of the participants had also been diagnosed with postural orthostatic tachycardia syndrome, and one was confirmed to have a herniated disc.

In the reflexive thematic analysis, two main themes and five subthemes were generated from the participants’ narratives (see table 1).

**Table 1:** Themes and subthemes

Theme	Subthemes
I am stumbling in the dark: Living with unpredictability and limitations	How do I live and function now? Who am I now?
I need you to trust me and help me: Navigating responses from others to find support	Stop being sceptical! Just try to help me! Accept and validate me!

### I am stumbling in the dark: Living with unpredictability and limitations

The first theme, *I am stumbling in the dark*, describes how participants experience long COVID symptoms and try to manage them and how the symptoms have affected various aspects of their daily lives, as well as their view of themselves. The subtheme *How do I live and function now?* highlights how unpredictable long COVID is experienced by the participants, even if they observe that symptoms often, but not always, worsen with exertion. It also illustrates how long COVID drastically limits them in their everyday lives. In the subtheme *Who am I now?*, the CYP describe how their perception of themselves has changed and how the effects of long COVID have affected their emotional well-being negatively.

#### How do I live and function now?

All participants described how they are trying to adjust their lives to their long COVID symptoms, which are irregular and difficult to predict. Elsa described how symptoms fluctuate in intensity:

[…] high heart rate, fever, headache, brain fog, altered breathing patterns, and I’m probably forgetting a lot of things, but it’s been ongoing the whole time, although it comes in waves. Some months are better in some ways, and some are worse in others.

The unpredictability of symptoms, like Elsa described, led to many CYP saying that they did not know how they would feel from one day to the next. Similarly, they were uncertain whether symptoms ever will disappear completely. However, while the symptoms were unpredictable, participants repeatedly described their symptoms as triggered by physical or mental exertion. Also here, it was unclear to the CYP how activity would influence their symptoms because symptoms rarely appeared immediately after exertion. Martin talked about how difficult it was to link the exertion to the symptoms:

That’s the weirdest part — we’re a bit unsure. It’s just, like, sometimes it comes, sometimes it doesn’t. […] And it’s kind of hard to know what triggers it when it happens the next day. If you do something one day, it’s usually not just that one thing. […] Even if you just go out to a restaurant, you don’t know if it was all the people you met, the kind of food you ate, where you were, or the car ride. It’s hard to know.

The participants described recurring strategies to manage the unpredictable symptoms, by conserving energy, planning and prioritising.

Despite difficulties in predicting worsening of symptoms, all participants described that it was too exhausting to attend full school days. Natalie explained “I can’t be there the entire day because then I’m bedridden the next day. My body can’t handle it.” Various sensory inputs in the school environment exacerbated the symptoms for several of the CYP. Sarah said “It’s difficult, partly because in school there are a lot of people, a lot of movement, and a lot of noise. And that makes me very tired and dizzy… and things like that.” Although school was experienced as challenging, most participants chose to prioritise it, consuming most of their available energy there. Anna exemplified her strategies of planning and prioritising in relation to school:

It’s been a lot of, really a lot of planning. Planning and prioritizing what I want to focus on. Okay, I want to focus on the important subjects—maths, physics, and those. And really try. What can I handle? What is worth doing? Trying to save my energy for the whole week.

As school consumed so much of the participants’ energy, activities outside school hours were difficult. In relation to her talk about school above, Anna shared that she has not “done anything beyond school. I haven’t had the energy to do anything when I get home, like meeting friends or anything.” Like Anna, all CYP described a change in their social lives, with most stating that physical social interactions were exhausting and could trigger symptoms. Consequently, interactions with peers had diminished or ceased entirely. Digital interactions were mentioned by a few CYP as an alternative to physical socialisation. A couple of participants described how their long COVID had resulted in having fewer social contacts than before. Sarah told us that she had friends before, but that her long COVID led to her losing them: “I tried and tried to keep in touch with them. But no one reaches out to me anymore, and I just haven’t felt like they’ve been very understanding.” In other words, many CYP lost the social support from friends that they potentially needed to cope with the changes long COVID brought about.

In addition, most participants had to quit their leisure activities after developing long COVID. Elsa explained how she did sports and creative hobbies but “had to quit everything” after developing long COVID. Participation remained difficult, even in adapted leisure activities. Walther explained: “And like, at [sport] for example, I skipped the warm-up so I wouldn’t have to run and stuff. But pretty much every time after [sport], I get sick the next day with a headache.” This led to many being inactive in their leisure time because of their new limitations.

This subtheme illustrates how living with limiting and unpredictable symptoms of long COVID has completely changed CYP’s lives and that their new reality contrasts sharply with their lives before falling ill. All participants expressed a desire to do significantly more than they were currently able to, and the subtheme shows how participants have been forced to find strategies to navigate both symptoms and limitations of living with long COVID. Balancing and planning were described as necessary and helpful, but made the participants feel more restricted in relation to hobbies and interactions with peers.

#### Who am I now?

As described above, the symptoms of long COVID and the strategies to handle them led the CYP to feel that “everything has changed”, as Elsa put it. In describing these life-altering consequences, it became clear how they shaped participants’ self-perception, both in relation to their pre-COVID selves and to their peers. Many experienced negative effects on their well-being.

Like others quoted above, Sarah described how her ability to function was profoundly affected by her long COVID symptoms. She said: “It’s very hard to function. As I said, I haven’t been able to attend school for a long time, I can’t help much with the [pets], I can’t hang out with friends, and I can’t do much at all.” She explained that her academic performance had been central to her identity before developing long COVID: “I’ve always been a person who bases my worth on how I can perform, and when I suddenly couldn’t perform, I valued myself much less.” According to Sarah, the changes caused by long COVID affected not only her daily life but also eroded the foundation of her self-worth and identity. Other CYP similarly struggled to lower their performance standards, as they could no longer manage schoolwork at the same level, which affected how they evaluated themselves.

In addition, not being able to hang out with friends or continue doing sports had implications for the CYP’s identities. For example, Anna shared: “I played [sport]. It was a big part of my identity.” Because the difference of who they were before COVID and now with long COVID was so substantial, several participants expressed a desire for everything to return to normal. When asked how he felt, Martin said “of course, it’s sad, and I want to go back to how things were before”, illustrating the longing to be able to become who one was before COVID. A similar wish was voiced by several participants.

The CYP not only compared their own lives before and after long COVID but also compared themselves with same-aged peers. The feeling of being different from others was recurring. Several participants described feeling frustrated by not being able to do what others their age could do, such as “traveling, working, or planning their studies, while I can’t do anything” as Anna put it. Feelings of shame and the desire to hide one’s condition were also described by some CYP. Natalie said:

I don’t want everyone to know either. Because I don’t like when people see me, like, in pain or something. I was the girl in the wheelchair at school—I had to have it in [grade]—and I don’t want to be that again. So, I decided that no one else should know except my friends that I need it or something like that.

A few participants blamed themselves for not being able to handle their condition and its impact on themselves and others. At times, this self-blame was expressed as anger. Natalie explained this and said: “I kind of get mad at myself for not being able to control it, but then I also get sad because I can’t.” Most of the young people also expressed guilt towards their parents, who had to adjust and make sacrifices from the onset of their illness. Anna expressed concern and guilt about how her symptoms affected her parents’ well-being:

Sometimes, because I’ve been so tired, I haven’t been able to see how much it has affected them. So, I can feel a bit guilty about that sometimes. They’ve done so much; they probably don’t feel great either. They’re affected too—they get worried.

This illustrates how the CYP, in addition to dealing with severe and limiting symptoms, also deal with guilt and self-blame. Most participants highlighted how long COVID negatively affected their emotional well-being, and one participant shared that she had experienced suicidal thoughts because of long COVID.

This subtheme illustrates how long COVID restricted the CYP to live a life aligned with their interests and self-image. Placed in a new position as chronically ill, many CYP shared that they felt distressed about what they could no longer do or be, and all they wanted was to get help to deal with, and hopefully improve, their symptoms.

### I need you to trust me and help me: Navigating the responses from others to find support

The second theme, *I need you to trust me and help me*, highlights the responses and (un)supportive actions of others. The participants often described encountering distrust and lack of understanding in healthcare settings and schools and among friends, as illustrated by the subtheme *Stop being sceptical!* However, many also described that even when professionals in schools or healthcare are not sceptical, their lack of knowledge could lead to frustrating interactions, as described in the subtheme *Just try to help me!* Finally, the subtheme *Accept and validate me!* shows the positive experiences of being seen and heard, as well as encountering professionals knowledgeable about long COVID.

#### Stop being sceptical!

Most participants told us about experiences of not being understood or taken seriously by healthcare providers, school staff and peers. Elsa described how healthcare professionals doubted the diagnosis and said: “I’ve met many doctors who don’t believe long COVID exists.” Similarly, Natalie shared how scepticism within healthcare led to degrading treatment: “I had nurses physically pull me out of bed because they didn’t believe me. They took away my wheelchair and said I could walk and that I was lying.” The CYP asked how adults who are supposed to help can act like this. Natalie further emphasised the importance of trusting patients’ accounts. If scepticism persists, the aim of helping the CYP should still be in focus, not dismissal:

More confidence in — like, trust us. No child can fake paralysis… You can’t fake it because you would get caught. […] So, if a child says they’re paralyzed and shows symptoms, believe them. […] And if you think they’re faking, figure out why instead of just dismissing them. Find the underlying issue. […] Listen to children because we know our own bodies.

Similar scepticism had been experienced by the CYP in schools and among peers. For example, Linus described facing disbelief from the staff in school: “At my previous school, I didn’t get much help. They didn’t believe me much and didn’t listen.” Martin also described feeling resistance from the school to do any accommodations at all: “They didn’t even want to give me access to school resources at home. They wanted me to come to school in a condition where it was completely impossible without making myself a hundred times worse.” Several CYP described how a directly negative approach from schools meant not receiving the support they needed, which made all the challenges they experienced even more burdensome.

Similarly, several CYP described dismissive comments from peers. Sarah, for example, described how a classmate questioned her long COVID by comparing it to their own experiences: “A friend said to me, ‘I also get headaches and dizziness, but you get to go home from school—why is that?’”. She described how she felt unseen when her illness was compared with more ordinary episodes of headaches. Sarah further went on to explain how others’ recurring questioning and scepticism caused her to doubt herself:

It was so hard because I didn’t feel believed by anyone. I kept thinking there was something wrong. It felt like people didn’t really believe me, and then I started to not believe in myself as much. I find that very difficult. Multiple times a day, I think about it. It feels like I wasn’t believed by the people I had hoped would believe me.

The participants’ stories revealed disappointment with healthcare professionals and teachers, who were supposed to help but instead showed doubt. This questioning from others was perceived as leading to self-doubt, frustration and despair, and the main message from the participants was that professionals have to stop being sceptical.

#### Just try to help me!

Even when participants did not experience explicit scepticism or doubt, many professionals in schools and in healthcare just did not know how to help, which created frustration and disappointment.

The participants were frustrated when healthcare could not explain what was wrong and sent them home to wait and return later. Most CYP said they had sought healthcare, but that it did not lead anywhere. Anna described how disheartening it was to go without explanation:

I think we sought care in March, a few months after, for my fatigue, and then I got the response that I should wait a year and then come back to see if it had gotten better. So that was the answer I got, and I remember it so clearly. It was like: okay, what am I supposed to do now? I don’t feel well, I want help. They did blood tests, and everything looked normal, so I was sent home again.

Some participants experienced that they and their parents were more knowledgeable than the healthcare practitioners and that they had to guide them on how to proceed with the assessment. Elsa said that she had to push to undergo the same examinations as other long COVID patients, but instead had to repeat the same tests multiple times:

Yes, a tilt test. You’re tilted, like. And we’ve been trying to get that for three years . … They usually just do a resting ECG and take blood tests. And they do that every time I go somewhere. And it’s always normal. We usually tell them it’s almost pointless to take it because it’s going to be normal. And it always is. So, it’s not very helpful.

In addition to Anna’s and Elsa’s dissatisfaction with healthcare, Linus described that he had been given an intervention that was not suited to his needs: “now I go to this [physiotherapy intervention] that I think is completely unnecessary. It’s more for those who are afraid to move.” As such, Linus felt that the intervention missed the target and thus became irrelevant for him. These are some of the many examples when CYP did not feel supported by healthcare, even when clinicians were not dismissive.

Similarly, several CYP described how schools also did not know how to support them, as there was no previous knowledge or experience of long COVID. Sarah described how her school psychologists tried to help here in a way that did not become supportive:

For a while, I went to the school psychologist, and we tried to come up with something, but it was a lot of: ‘do you have any ideas?’ And they seemed to have never encountered anything like this before. And it was hard for me to come up with something because I’d never experienced this before.

Most CYP said that they and their parents had to push schools to make accommodation. However, while several participants felt dismissed, others also had positive experiences where they felt that the school listened and tried their best. The participants expressed empathy towards the lack of knowledge of what to do, as long COVID is a new condition. However, they still had to deal with their condition, and when parents, schools and healthcare professionals did their best to help, it made a lot of difference.

#### Accept and validate me!

In contrast to scepticism and a lack of knowledge, many participants also described experiences where they felt believed and validated. They emphasised how much it meant to have someone who believed in their experiences and their descriptions of symptoms and helped to find interventions and accommodations which were truly supportive, which, in turn, became validating.

Many CYP expressed gratitude towards their parents for believing them. Martin appreciatively described how his parents were attentive to his signals and supported him in managing both his health and school:

They know me and recognize when I’m unwell […]. My parents have been incredibly supportive. They know me, they listen to me a lot and notice when I’m feeling worse. When that happens, they ensure I take it easy and don’t get worse. They’ve also fought hard with the school to ensure I get what I need.

Even though the participants’ parents did not always know how to help them, all participants testified that their parents did their best. Anna said “Yeah, they’ve helped me a lot, even though it’s not easy to help me because it’s hard to know how I feel. Like, I don’t even really know how I am.” Sarah described how her parents helped her with various things at home: “They support me in so many ways. They help me remember things. I can’t manage to put food on my plate, so they help me with that.” Emotional support was also mentioned. Anna shared: “They’ve helped support me when I’ve been sad or angry.” As such, all CYP were thankful for their parents.

Most participants also had some examples of empathetic encounters with school or healthcare staff. Natalie described a healthcare meeting that underscored the value of being heard and believed. She said: “I had a fantastic doctor who believed in me and conducted many tests. She couldn’t help me, unfortunately, but just the fact that she listened meant so much.” Like Natalie, several CYP emphasised that what matters most is not what healthcare professionals do, but how they do it and how they listen, ask questions and conduct assessments.

Meeting healthcare professionals with expertise in long COVID was described as particularly meaningful. Elsa described a team that helped her:

They had some rehab program . I thought it was super helpful because I got an occupational therapist and a physiotherapist. And someone else, and they were a small team. I got a lot of help there. I went there twice a week and did some exercise at my level.

Anna also described how visiting a specialised clinic felt like a major change, with the staff adapting the assessment based on knowledge about long COVID. She said: “The staff at that clinic expected me to be tired and gave me lots of breaks. Even though I underwent many tests, they adjusted for me: ‘You’re tired now, right? Take a 20-minute break.” Martin shared a similar experience, saying his “care improved because I was treated by people who understand long COVID and know from others’ experiences what might help or worsen the situation.” These descriptions show that getting support from knowledgeable clinicians felt truly helpful.

In schools, some participants noted a connection between being heard and the support they received. Elsa explained that she was satisfied with several accommodations made by her school, which reduced physical strain and sensory input. She also received adapted meals and access to a recovery room. She described: “I got to sit alone in a room for tests. Someone read the tests aloud to me, and they had rooms I could go to for breaks”. She added “Every teacher was informed about my situation, so many of them could adapt assignments, or if there were certain assignments, I only needed to do the most necessary ones.” Her narrative of her school contrasted sharply to those CYP who received no or little support. As such, Elsa’s story also highlights what a difference schools can make for CYP with long COVID.

This subtheme illustrates that since the CYP in this study were accustomed to having their experiences questioned, validation was highly significant. Even when healthcare could not cure their long COVID, listening, caring and doing what was possible mattered immensely.

## Discussion

The aim of this study was to give voice to CYP living with long COVID and understand their experiences from a holistic perspective. The themes ‘I am stumbling in the dark: Living with unpredictability and limitations’ and ‘I need you to trust me and help me: Navigating responses from others to find support’ show that living with long COVID involved managing fluctuating symptoms, uncertainty and identity disruption, while also seeking recognition, validation and practical support from healthcare, school and parents.

This study adds to the limited qualitative research on CYP’s own experiences of long COVID.^13^,^18^ Consistent with previous findings, the subtheme *How do I live and function now?* emphasises how participants’ experiences of unpredictable symptoms made it difficult to anticipate daily functioning. Still, many reported that both physical and mental exertion could trigger or worsen symptoms, often with a delay, consistent with post-exertional malaise, a symptom affecting over half of long COVID patients.^25^ This also aligns with parental reports of how symptoms, such as headaches and brain fog, can follow days with high activity levels.^19^ The drastic changes in the participants’ everyday lives, including their social lives, and the required constant navigation of symptoms are important to analyse from a developmental perspective. Managing such uncertainty and continually adjusting activity levels may place overwhelming demands on self-regulation and responsibility. This is particularly salient in adolescence, a developmental phase characterised by increasing autonomy from caregivers and identity formation.^11^

In the subtheme *Who am I now?*, the participants described how long COVID disrupted developmental processes, leading to increased dependence on parents and loss of capacities central to their sense of self (eg, participation in school and leisure activities). The findings align with conceptual work on adolescent-onset chronic illness describing an ongoing identity renegotiation when everyday roles and future plans become uncertain.^26^ Although parent–CYP relationships were described as supportive and trusting, participants expressed guilt that their parents now needed to care for them more and advocate for them in interactions with healthcare and school. While parental guilt has been reported,^14^ the guilt felt by CYP towards their parents is a novel finding, possibly facilitated by the fact that most interviews were conducted without parents present.

This sense of guilt may also suggest that participants and their families were left to shoulder much of the burden of the illness themselves, highlighting the limited recognition and support available in healthcare and schools. This is particularly relevant given the broader contestation surrounding long COVID, both regarding its definition and the support that healthcare systems and society are expected to provide, including in Sweden where long COVID in children remains highly contested.^27^ In line with previous long COVID research,^8 14 16 17 28^ the subthemes *Stop being sceptical!* and *Just try to help me!* illustrate how the participants struggled both to be believed and to obtain adequate support from healthcare services and schools. These subthemes should not only be considered as simple requests for better communication, but may also be interpreted as accounts of negotiations of credibility and legitimacy in encounters with healthcare and education systems. Encountering scepticism from professionals was described as adding stress beyond the illness itself. In a similar vein, the accounts also reflect the added emotional and relational work involved in living with an invisible condition, where limitations may be questioned by others and CYP must continually manage disclosure and credibility.^29^

Research on identity formation in CYP living with a chronic illness during adolescence has highlighted the importance of accepting and adapting to the illness to move forward.^30^ However, such adaptation is not an individual process alone but is shaped by whether CYP encounter support. In the subtheme *Accept and validate me!* the CYP described how small acts of affirmation and adaptations made them feel seen and heard. Being met with acknowledgement and empathy from healthcare professionals is fundamental to feeling safe and supported. Previous research has shown this in relation to other chronic illnesses during adolescence,^26^ as well as in other studies on long COVID in CYP^15 17^ and adults.^28^ The present findings suggest that validation may be especially important in the context of long COVID, where uncertainty and contestation can otherwise leave CYP to manage too much of the burden on their own.^27^

### Implications for practice

Our findings suggest several implications for healthcare and educational systems. In clinical encounters, CYP emphasised the importance of being believed, taken seriously and treated respectfully, particularly given uncertainty and symptom variability. Clinicians should prioritise validation practices (acknowledging uncertainty while affirming symptom reality), avoid dismissive explanations and offer concrete guidance on managing fluctuating capacity. Given participants’ accounts of identity loss and guilt, psychosocial support that addresses dependence, stigma and self-concept may also be beneficial.

Schools play a central role in enabling participation, something all participants wished for. Accommodations should be individualised, flexible and reviewed over time (eg, reduced workload, flexible attendance/deadlines, rest breaks, remote participation when needed).^15 31^ At a system level, accounts indicate a need for improved coordination between healthcare and schools.^31^ Written recommendations or shared care plans describing functional impacts and suggested accommodations may reduce the burden placed on families to repeatedly legitimise the condition. Where feasible, specialised or multidisciplinary services with knowledge of paediatric long COVID may support consistent advice, continuity and integrated medical–psychosocial–educational support.

### Strengths and limitations

The strengths of this study include a fairly balanced gender distribution among participants and the fact that most interviews were conducted without parents. It is unclear whether parental presence in two interviews restricted disclosure of sensitive information; still, we wanted to respect the participants’ wishes to include a parent as a passive support.^32^

The relatively extensive interviews (mean duration of 51 min) generated a rich dataset. Digital interviews yield high-quality data and are particularly suitable for younger participants.^33^ For CYP with long COVID, they can also reduce physical strain and accommodate fluctuating energy levels, thereby supporting participation. However, the hourlong format may have limited involvement of CYP with very severe long COVID.

Participants were recruited through purposive sampling based on predefined inclusion criteria. However, because recruitment occurred via an open call, elements of convenience sampling were also present, as in many qualitative studies. Recruitment was challenging, consistent with other long COVID research.^13^ While the dataset was judged adequate for our reflexive thematic analysis aims based on the richness of the interviews and iterative development of coherent themes across the dataset, we do not claim thematic completeness.^21^

Thus, several factors should be considered when results are being transferred to other contexts. Recruiting through a patient organisation may have influenced who volunteered, potentially leading to an overrepresentation of CYP with higher symptom burden, greater parental engagement and higher health literacy. The Swedish context, in which all healthcare services are free of charge up to at least the age of 18 and all education is provided free of charge, frames the conditions under which these CYP live. However, governance of both healthcare and education is decentralised in Sweden. Consequently, the care and interventions received by the participants in our study can be assumed to vary.

## Conclusions

When interviewed in depth, CYP living with long COVID described that they have an unpredictable and disabling condition that restricts them from living as they did before and as their peers of the same age do. Long COVID is experienced as affecting all major areas of life, including identity and independence. The CYP have met dismissiveness and lack of knowledge but expressed a strong desire for educational and healthcare personnel to validate their experiences and provide meaningful support. In schools, this means implementing support already existing for other groups of students. In healthcare, it means providing an empathetic and validating approach that implements assessments and interventions that do exist and help to deal with the uncertainty of living with long COVID.

## Data Availability

No data are available.
