# Supplementary material for: “It’s sad, and I want to go back to how things were before”: a qualitative study of young people’s experiences of living with long COVID
Source: BMJ Paediatr Open. 2026 May 11;10(1):e004167. doi: 10.1136/bmjpo-2025-004167 (PMC13182309; doi:10.1136/bmjpo-2025-004167)
Supplement: Supplementary file 1 [file bmjpo-2025-004167.reviewer_comments.pdf]

PEER REVIEW HISTORY

BMJ Paediatrics Open publishes all reviews undertaken for accepted manuscripts. Reviewers are asked to complete a checklist review form and are provided with free text boxes to elaborate on their assessment. These free text comments are reproduced below.

ARTICLE DETAILS

**TITLE (PROVISIONAL)**	"It's sad, and I want to go back to how things were before": A qualitative study of young people's experiences of living with long COVID
**AUTHORS**	Järvholm, Kajsa<br>Bygdell, Maria<br>Dreifaldt, Johan<br>Åkesson, Elin<br>Lundberg, Tove

VERSION 1 – REVIEW

**REVIEWER NAME**	Wasilewski, Marina B.
**REVIEWER AFFILIATION**	
**REVIEWER CONFLICT OF INTEREST**	No Conflict of Interest
**AI DISCLOSURE**	No, I have not used AI
**DATE REVIEW RETURNED**	02-Dec-2025

**GENERAL COMMENTS**	Thank you for the opportunity to review this manuscript. It covers a very important and timely topic, and the authors are commended on spotlighting the voices of CYPs, who are largely underrepresented in current Long COVID research. I have made some suggestions below that I hope will strengthen the manuscript.<br><br>- The design description doesn't really outline a qualitative study design. Was qualitative description used? Phenomenology?<br>-The age range for participants needs further justification. Why not include CYP younger than 12 (especially if parents could be present to support the interview)? Why were CYP up to 21 included when adult age is typically 18+?<br>-Good detail around the analysis process and researchers involved<br>-The themes are interesting and well-described. They add to our understanding of how long COVID is uniquely experienced by CYP.<br>-Given the small sample size (n=7), the authors need to critically reflect on data quality and saturation.

**REVIEWER NAME**	Pinzón-Segura, María
**REVIEWER AFFILIATION**	Universidad Nacional de Colombia
**REVIEWER CONFLICT OF INTEREST**	No Conflict of Interest
**AI DISCLOSURE**	I used OpenAI's ChatGPT (based on the GPT-4 architecture) as a language support tool during the preparation of my review. Its role was limited to assisting with tasks such as refining the language, clarifying and organizing my thoughts, and ensuring the text adhered to a clear and structured format. All substantive evaluations

	and the ultimate conclusions expressed in the review reflect my own critical assessment, and the AI served solely as a writing aid.
**DATE REVIEW RETURNED**	01-Mar-2026

**GENERAL COMMENTS**	Thank you for the opportunity to review this thoughtful and important qualitative study exploring the lived experiences of children and young people (CYP) with long COVID. The manuscript addresses a clear gap in the literature by foregrounding CYP voices independent of parental perspectives and contributes valuable insights into identity disruption, educational impact, and experiences of validation and invalidation within healthcare and school systems.<br><br>Overall, the study is well designed, methodologically sound, and clinically relevant. The use of semi-structured interviews and critical realist thematic analysis is appropriate to the stated aim of giving voice to CYP. The findings are coherent, clearly presented, and supported by illustrative quotations. The manuscript aligns well with BMJ Paediatrics Open priorities in patient experience and child-centred care.<br><br>However, several areas would benefit from strengthening before publication.<br><br><br>Major points<br><br>1. Reflexivity and researcher positioning<br><br>The manuscript describes the research team's professional backgrounds, but reflexive positioning in relation to long COVID and CYP care is limited. Given the qualitative approach and the team's clinical and advocacy roles, a brief reflexivity statement would strengthen transparency and analytic credibility (e.g., assumptions, prior involvement with long COVID services or patient organisations, potential interpretive influences).<br><br>2. Sample size and saturation<br><br>The study includes seven participants. While this can be appropriate in qualitative work with hard-to-reach populations, the manuscript does not explicitly address data sufficiency or saturation. Please clarify how the team judged that the dataset was adequate to support the themes, or acknowledge limits to thematic completeness.<br><br>3. Transferability and contextualisation<br><br>The findings arise from a Swedish healthcare and educational context with recruitment partly via a patient organisation. The discussion would benefit from more explicit consideration of transferability, including how contextual features (e.g., healthcare access, school accommodations, parental advocacy structures) may shape experiences and applicability to other settings.<br><br>4. Analytic depth<br><br>The analysis is described as descriptive thematic with a "giving voice" orientation. While appropriate, some sections of the discussion remain relatively descriptive. The manuscript could be

	strengthened by deeper engagement with relevant conceptual literature (e.g., adolescent identity disruption in chronic illness, stigma in contested conditions, invisible disability) to enhance interpretive contribution.<br><br>5. Implications for practice and systems<br><br>The study has clear implications for healthcare and educational systems, yet these remain somewhat implicit. Consider expanding the implications section to articulate specific recommendations for clinicians, schools, and service organisation (e.g., validation practices, coordinated school-healthcare accommodations, specialised services).<br><br>Minor points<br>• Please clarify whether thematic saturation or information power was considered during analysis.<br>• Consider noting potential sampling bias related to recruitment through a patient organisation (severity, parental engagement, health literacy).<br>• A brief statement on diversity (e.g., socioeconomic or ethnic background) would help readers assess transferability.<br>• Some discussion paragraphs could be condensed to avoid repetition.<br><br>Conclusion<br><br>This is a valuable and timely qualitative contribution that centres CYP perspectives on long COVID. With strengthened reflexivity, contextualisation, and analytic depth, the manuscript will make a meaningful contribution to paediatric and health services literature.<br><br>Recommendation: Accept with major revisions.

VERSION 1 – AUTHOR RESPONSE

Reviewer 1
Thank you for the opportunity to review this manuscript. It covers a very important and timely topic, and the authors are commended on spotlighting the voices of CYPs, who are largely underrepresented in current Long COVID research. I have made some suggestions below that I hope will strengthen the manuscript.
Dear Dr. Wasilewski, we are very grateful for your comments and thoughtful suggestions!
1. The design description doesn't really outline a qualitative study design. Was qualitative description used? Phenomenology?
Thank you for this comment. We agree that our original wording did not clearly specify the qualitative study design/approach. We have revised the Design section to explicitly describe the study as an interpretive qualitative interview study, underpinned by a critical realist perspective and analysed using reflexive thematic analysis (Braun & Clarke).<br><br>*We now write on p. 5:*<br>*To explore and give voice to the lived experiences of CYP with long COVID, we conducted an interpretive qualitative interview study. Data were generated through semi-structured, open-ended interviews and analysed using reflexive thematic analysis.* [21] *The study was grounded in a critical realist perspective, which views participants' accounts as meaningful descriptions of experience shaped by material, social, and contextual conditions.*<br><br>*Data collection was planned with common long COVID symptoms, such as fatigue, in mind. Participants could therefore choose between digital or in-person interviews, and they were not*

asked to provide feedback on transcripts or analyses, a procedure sometimes used in qualitative research.
2. The age range for participants needs further justification. Why not include CYP younger than 12 (especially if parents could be present to support the interview)? Why were CYP up to 21 included when adult age is typically 18+?
Thank you for this comment. We have now clarified and justified the chosen age range in the manuscript. The lower age limit of 12 years was set because we aimed to ensure that participants could meaningfully engage in an interview without relying on extensive parental support. Although two participants chose to have a parent present, this was not planned in advance and does not alter the rationale for the lower age boundary.<br><br>*Including young people up to 21 allowed us to capture their perspectives on the long-term impact of an illness that began in adolescence, even if they had since transitioned to adult services. We have now added this explanation to the manuscript.*<br><br>*We now write on p. 6:*<br>*The primary inclusion criterion was long COVID onset before the age of 18 years. Long COVID was defined according to the WHO criteria, namely persistent symptoms lasting at least two months and occurring at least three months after acute COVID-19.22 CYP aged 12–21 years were eligible. The lower age limit was set to increase the likelihood that participants could engage meaningfully and independently in the interviews, while the upper limit allowed inclusion of those who developed long COVID in late adolescence.*
3 & 4. Good detail around the analysis process and researchers involved.<br>The themes are interesting and well-described. They add to our understanding of how long COVID is uniquely experienced by CYP.
Thank you for these positive comments on both the analytic description and the thematic findings, and for noting their contribution to understanding how long COVID is experienced by CYP.
5. Given the small sample size (n=7), the authors need to critically reflect on data quality and saturation.
Thank you for raising this important point. We agree that addressing data quality is important and has now included a reflection on that in the manuscript (see data analysis and the limitation-section in the discussion).<br><br>*As we did a thematic analysis, we have followed Braun and Clarke's critical recommendation not to use "saturation" in this article as it is a concept from grounded theory and "do not always translate (well) to, or cohere with, TA." (Braun & Clarke, 2022, p. 337). Given limited space, we do not provide an extended methodological discussion of saturation debates; however, we have added a brief clarification on how we judged data adequacy for our analytic claims.*
Reviewer 2
Thank you for the opportunity to review this thoughtful and important qualitative study exploring the lived experiences of children and young people (CYP) with long COVID. The manuscript addresses a clear gap in the literature by foregrounding CYP voices independent of parental perspectives and contributes valuable insights into identity disruption, educational impact, and experiences of validation and invalidation within healthcare and school systems.<br><br>Overall, the study is well designed, methodologically sound, and clinically relevant. The use of semi-structured interviews and critical realist thematic analysis is appropriate to the stated aim of giving voice to CYP. The findings are coherent, clearly presented, and supported by illustrative quotations. The manuscript aligns well with BMJ Paediatrics Open priorities in patient experience and child-centred care.<br><br>However, several areas would benefit from strengthening before publication.
Dear Dr. Pinzón-Segura, we are very grateful for your comments and thoughtful suggestions!
1. Reflexivity and researcher positioning<br>The manuscript describes the research team's professional backgrounds, but reflexive positioning in relation to long COVID and CYP care is limited. Given the qualitative approach and the team's clinical and advocacy roles, a brief reflexivity statement would strengthen transparency and analytic credibility (e.g., assumptions, prior involvement with long COVID services or patient organisations, potential interpretive influences).

Thank you for this important comment. Prompted by your comment, we discussed whether our thematic analysis was reflexive or not. After going back to Braun and Clarke's texts, we do think we have followed their recommendations on reflexive thematic analysis (RTA, and not only code-book TA). We have thus restated this analysis as an RTA and also explicitly included our reflexivity (e.g. our use of our professional backgrounds and our knowledge on long Covid and health care) more clearly in the data analysis section.<br><br>*We now write on p. 8:*<br>*Throughout the analysis, we engaged in reflexive discussions and considered alternative interpretive possibilities. In keeping with our aim of "giving voice", we prioritised participants' meanings and language in the development and presentation of themes. Still, we acknowledge that theme development is an interpretive process also shaped by our clinical and advocacy engagements. For example, all involved researchers share the position that long COVID is a real and significant condition and that CYP deserve high quality care where their symptoms are taken seriously.*
2. Sample size and saturation<br>The study includes seven participants. While this can be appropriate in qualitative work with hard-to-reach populations, the manuscript does not explicitly address data sufficiency or saturation. Please clarify how the team judged that the dataset was adequate to support the themes, or acknowledge limits to thematic completeness.
Thank you for this comment. Given the hard-to-reach nature of CYP with long COVID and the participation burden associated with post-exertional symptom exacerbation/marked fatigue, seven in-depth interviews constitute an ethically feasible and information-rich dataset. We judged adequacy through iterative theme development and refinement across the full dataset, and we now acknowledge limits to thematic completeness in the manuscript. In line with Braun and Clarke, we did not use "saturation" as a criterion for RTA (Braun & Clarke, 2022). Please also see response to Reviewer 1's comment 5.
3. Transferability and contextualization<br>The findings arise from a Swedish healthcare and educational context with recruitment partly via a patient organisation. The discussion would benefit from more explicit consideration of transferability, including how contextual features (e.g., healthcare access, school accommodations, parental advocacy structures) may shape experiences and applicability to other settings.
Thank you for this valuable comment. We agree that the Swedish healthcare and educational context, as well as our recruitment strategy via a patient organisation, have implications for the transferability of our findings. In the revised manuscript, we have added a dedicated paragraph in the Discussion where we explicitly address these contextual factors.<br><br>*We now write on ps. 23-24:*<br>*Thus, several factors should be considered when results are being transferred to other contexts. Recruiting through a patient organization may have influenced who volunteered, potentially leading to an overrepresentation of CYP with higher symptom burden, greater parental engagement, and higher health literacy. The Swedish context, in which all healthcare services are free of charge up to at least the age of 18 and all education is provided free of charge, frames the conditions under which these CYP live. However, governance of both healthcare and education is decentralized in Sweden. Consequently, the care and interventions received by the participants in our study can be assumed to vary.*
4. Analytic depth<br>The analysis is described as descriptive thematic with a "giving voice" orientation. While appropriate, some sections of the discussion remain relatively descriptive. The manuscript could be strengthened by deeper engagement with relevant conceptual literature (e.g., adolescent identity disruption in chronic illness, stigma in contested conditions, invisible disability) to enhance interpretive contribution.
Thank you for this suggestion. We have strengthened the discussion by engaging more explicitly with conceptual literature to deepen interpretive contribution, including work on adolescent identity disruption in chronic illness, stigma in contested conditions, and invisible disability. These lenses help interpret our themes as involving not only symptom management but also identity renegotiation and negotiations of legitimacy in healthcare and school encounters.<br><br>*Please see the revised discussion on pages 20-24.*

5. Implications for practice and systems<br>The study has clear implications for healthcare and educational systems, yet these remain somewhat implicit. Consider expanding the implications section to articulate specific recommendations for clinicians, schools, and service organisation (e.g., validation practices, coordinated school-healthcare accommodations, specialised services).
Thank you for this insightful comment. In the revised manuscript, we have gathered all content related to implications and structured it more clearly within the discussion. We now include two dedicated paragraphs where we explicitly elaborate on the implications for clinical practice and schools, providing a more transparent and actionable synthesis of the study's contributions.<br><br>*We now write on ps. 22-23:*<br>*Implications for practice*<br>*Our findings suggest several implications for healthcare and educational systems. In clinical encounters, CYP emphasised the importance of being believed, taken seriously, and treated respectfully, particularly given uncertainty and symptom variability. Clinicians should prioritise validation practices (acknowledging uncertainty while affirming symptom reality), avoid dismissive explanations, and offer concrete guidance on managing fluctuating capacity. Given participants' accounts of identity loss and guilt, psychosocial support that addresses dependence, stigma, and self-concept may also be beneficial.*<br><br>*Schools play a central role in enabling participation, something all participants wished for. Accommodations should be individualised, flexible, and reviewed over time (e.g., reduced workload, flexible attendance/deadlines, rest breaks, remote participation when needed).15 31 At a system level, accounts indicate a need for improved coordination between healthcare and schools.31 Written recommendations or shared care plans describing functional impacts and suggested accommodations may reduce the burden placed on families to repeatedly legitimise the condition. Where feasible, specialised or multidisciplinary services with knowledge of paediatric long COVID may support consistent advice, continuity, and integrated medical–psychosocial–educational support.*
a. Please clarify whether thematic saturation or information power was considered during analysis.
See comment on information power and saturation (point 2).
b. Consider noting potential sampling bias related to recruitment through a patient organisation (severity, parental engagement, health literacy).
Thank you for this suggestion. We agree that recruiting through a patient organisation may have influenced who volunteered and, consequently, which experiences are most likely to be represented in the dataset. This recruitment pathway may have disproportionately reached families with higher symptom burden and/or greater parental engagement/advocacy and health literacy, thereby under-representing CYP who are less connected to such networks. We have added this as a sampling limitation and clarified implications for the scope and transferability of the findings.<br><br>*We now write on p. 23:*<br>*Recruiting through a patient organization may have influenced who volunteered, potentially leading to an overrepresentation of CYP with higher symptom burden, greater parental engagement, and higher health literacy.*
c. A brief statement on diversity (e.g., socioeconomic or ethnic background) would help readers assess transferability.
Thank you for this valuable comment. We agree that information on participants' socioeconomic or ethnic backgrounds would have helped contextualize the study further and supported readers in assessing transferability. Unfortunately, such data were not collected as part of the original study protocol, and we are therefore unable to provide those details.
d. Some discussion paragraphs could be condensed to avoid repetition.
In revising the manuscript, we have substantially reworked major parts of the discussion in response to your comment about analytic depth. As part of this process, we have also considerably reduced repetition of the results and condensed several paragraphs to improve clarity and flow.
Conclusion<br>This is a valuable and timely qualitative contribution that centres CYP perspectives on long COVID. With strengthened reflexivity, contextualisation, and analytic depth, the manuscript will make a meaningful contribution to paediatric and health services literature.
